# Predictive factors of post-operative atrial fibrillation after myocardial revascularization and its impact on mortality and morbidity

**DOI:** 10.1186/1749-8090-8-S1-P22

**Published:** 2013-09-11

**Authors:** LR Barbieri, AG Sousa, GMS Gerônimo, VLS Haddad, DFM Trompieri, MLP Sobral, GG Santos, NAG Stolf

**Affiliations:** 1 Hospital Beneficência Portuguesa de São Paulo, Brazil

## Background

Postoperative Atrial Fibrillation (POAF) is the most common arrhythmia in cardiac surgery. Its incidence ranges between 20-40%. The objective of this study was to determine the predictive factors of POAF that occurred in patients undergoing coronary artery bypass grafting (CABG), and the impact of this event on morbidity and mortality.

## Methods

This bi-directional and longitudinal cohort study was made with 3,010 patients undergoing CABG between June/2009 and July/2010, aged ≥ 18 years. We excluded 382 patients for chronic or paroxysmal atrial fibrillation, atrial flutter, or association of the surgery evaluated with other procedures.

## Results

The incidence of POAF was 12.4% (2302 progressed without POAF - Group I and 326 with POAF- Group II. Among the predictive factors for the occurrence of POAF, observed: age (Group I: 61.2 ± 9.38, group II: 66.8 ± 8.9, p <0.001); rate female: male ratio of 1:3; CRI (p=0.001), creatinine >/= 2.2mg/dl (p=0.002); COPD (p=0.001), ICC (p=0.004), EuroSCORE (p<0.001), blood transfusion (p<0.001), Peripheral Arterial Insufficiency (PAI) (0,011); emergency surgery/emergency (p= 0.006), postoperative stroke (p<0.001) and RI postoperatively (p <0.001). Patients with POAF had longer postoperative hospital stay (p<0.001), higher mortality at one year (p <0.001) and a higher rate of readmission within 30 days (p = 0.004).

## Conclusion

The age, male gender, CRI, COPD, peripheral arterial disease, creatinine >/= 2,2mg/dl, CHF, EuroSCORE, blood transfusion, emergency surgery/emergency (p= 0.006), postoperative stroke (p<0.001) and RI postoperatively (p<0.001) variables were independently predictive of POAF event. Due to the strong correlation with the increased length of hospitalization, mortality and re-hospitalization in these patients, we must implement strategies for POAF prophylaxis and prevention.

